# Psychometric properties of the Persian version of the Suicidal Ideation Attributes Scale among Iranian college students

**DOI:** 10.3389/fpsyt.2025.1437439

**Published:** 2025-03-31

**Authors:** Fatemeh Nazari, Amir Sam Kianimoghadam, Pardis Jamshidmofid, Mohammad Reza Norouzi

**Affiliations:** Department of Clinical Psychology, Behavioral Sciences Research Center, Shahid Beheshti University of Medical Sciences, Tehran, Iran

**Keywords:** suicide, validity, reliability, college students, Iran

## Abstract

**Background:**

Suicidality is a major global health problem and the second leading reason of mortality in 15–29-year-olds, and a growing subgroup of these youths are students. The present study aimed to evaluate the psychometric properties of the Persian version of the Suicidal Ideation Attributes Scale (SIDAS) among Iranian college students.

**Method:**

A total of 398 Iranian college students completed study measures online. The Beck Scale for Suicide Ideation, Beck Hopelessness Scale, and Connor–Davidson Resilience Scale were chosen for evaluating concurrent and divergent validities. The content validity index (CVI) index was calculated in order to check the face validity and content validity. Confirmatory factor analysis was performed to confirm the factors of the SIDAS. Finally, receiver operating characteristic (ROC) curves were constructed to assess scale sensitivity and specificity.

**Results:**

According to the findings, 286 (71.9%) of the participants were women. The average age of the sample was 23.9 years. According to this study, the Persian version of the SIDAS (SIDAS-P) demonstrated high internal consistency (a = 0.799), a unifactorial structure, a moderate correlation with the Beck Scale for Suicide Ideation (BSSI), and a negative moderate divergent validity with the Connor–Davidson Resilience Scale. Furthermore, the ROC curve analysis [area under the curve (AUC) = 0.909] indicated that the SIDAS-P could distinguish individuals with a high risk for suicidal behaviors (scores higher than 6.5 on the SIDAS are above the low risk for suicide).

**Conclusion:**

The findings support that the SIDAS is a valid and reliable online instrument for screening suicidal ideations in a population of non-clinical college students in Iran. To establish its clinical utility, further research should test its psychometric properties in clinical populations with different mental health problems.

## 1 Introduction

Suicidality is a leading global health problem (1), which has been defined as a death caused by self-injurious behavior with an intention to die due to this behavior (2). Suicide can happen at any point in life, and it is the second most common and in some countries the first cause of death among young individuals between the ages of 15 and 24 (3). It is estimated that one person every 40 seconds and approximately 800,000 individuals every year die by suicide (1). In the United States, suicide is the 10th main reason of death and accounts for almost 44,000 deaths each year. Although suicide attempts are less common in Islamic countries, evidence suggests that this number is increasing (4). The suicide rate in Iran is 5.3 per 100,000 people (5). According to a report, the suicide rate in different provinces of Iran ranges from 2.2 to 19.53 per 100,000 individuals (6).

Furthermore, the suicide rate is higher among medical students and physicians compared to the general population. According to reports from the Medical Council of Iran, 16 residents have tragically committed suicide in the past 12 months (7). In a study on medical students of Zahedan University of Medical Sciences, 17% of medical students had suicidal ideation, which is relatively high (8). Consistent with previous statistics, another study by Mohammadinia et al. revealed that 26.4% of nursing, midwifery, and medical emergency students in Iran had suicidal ideations (9). In addition, considering depressive symptoms as a risk factor for suicide, based on the results of a systematic review and meta-analysis study conducted in Iran (89 studies including a total of 33,564 Iranian students), approximately 50% of Iranian university students suffer from depression (10).

Suicide is a serious public health problem, with huge costs for psychiatric and other health services. In this regard, the hospitalization rate of people who attempted suicide is six to seven times higher than that of people who committed complete suicide. Suicide is also the seventh leading reason for lost years of potential life, ahead of liver disease, diabetes, and HIV (11). The impact of suicide on society can be very pervasive. Studies have shown that one person's suicide can affect an average of five close family members and up to 135 people (12).

The suicide spectrum encompasses suicidal ideation (SI), suicide plan (SP), suicide attempt (SA), and completed suicide (CS) (13). Despite the fact that most individuals who have suicidal thoughts do not commit suicide, suicidal thoughts may often be present before a suicide attempt (14). However, suicidal ideation is a prevalent precursor to the more severe consequences such as attempted suicide or complete suicide (15, 16). Therefore, in terms of prevention, this is an important issue that requires having reliable and efficient screening tools (17).

Although there are other scales that provide comprehensive information about the severity of suicidal ideation, since they must be administered by a clinically trained person and because of the long administration time, they are often not a suitable option for online or epidemiological surveys. This is while the Suicidal Ideation Attributes Scale (SIDAS) is a short five-item scale designed to be used online (18). The SIDAS is a new and short one designed to evaluate the intensity of suicidal ideation during the past month. It assesses five items: the frequency, controllability, and likelihood of suicide, as well as the distress and involvement with daily activities, which is related to such suicidal ideation (19).

According to the authors' knowledge, the psychometric properties of the SIDAS have not been evaluated in Iran. Considering that suicide is the second major reason for mortality in 15–29-year-olds (20) and a growing subgroup of these youth are students (21), the present study aimed to evaluate the psychometric properties of the SIDAS in college students of Iran. In the present study, we predicted that the results of statistical analysis for the SIDAS would indicate high internal consistency, appropriate convergent and divergent validity coefficients, and high test–retest reliability. Therefore, the appropriate efficacy of this tool for screening suicidal thoughts among the Iranian student population would be confirmed. Furthermore, considering the factor structure of the SIDAS, it was predicted that the original single-factor structure of the SIDAS would be repeated.

## 2 Method

### 2.1 Participants and procedure

After receiving ethical approval from the Research Center of Shahid Beheshti University of Medical Sciences (code number: IR.SBMU.RETECH.REC.1402.288), permission from the original authors (22) to use the SIDAS was obtained. As the first step, the questionnaire was translated into Persian by two clinical psychologists who were fluent in English (forward translation), and finally, a single version was extracted by a third researcher. Then, these two experts along with a linguist evaluated the translated questionnaire from a cultural and linguistic point of view, and the necessary changes were applied. Subsequently, an independent clinical psychologist who mastered the English language like a native speaker translated the questionnaire into English (backward translation). These steps were repeated until the inconsistencies between the original version and the translated one were resolved. Then, in order to evaluate the face validity of the questionnaire, the fluency and clarity of the items were evaluated by 10 Persian students and four clinical psychologists, with research and clinical backgrounds in the field of suicide, based on a 4-point Likert scale (0–3). The questionnaires were then pilot-tested and revised for clarity and fluency. The Beck Scale for Suicide Ideation, Beck Hopelessness Scale, and Connor–Davidson Resilience Scale were chosen for evaluating the concurrent and divergent validities. Considering that this questionnaire was designed to be implemented in online settings, the data were collected through a secured online platform. The link of the questionnaires was shared in groups of Tehran universities on social media between May 2023 and August 2023. As soon as students accessed the link, they were informed through an explanation that the survey data would be used anonymously and that there was no obligation for them to participate. As a result, only 398 students who were willing to participate in the study completed the questionnaires. Ultimately, a subgroup of 50 students was randomly selected to complete the questionnaire after 4 weeks. For the current study, the participants were included if they were students between the ages of 18 and 40. They would be excluded from the study if they answered the questionnaires in an incomplete or directed manner.

### 2.2 Measures

#### 2.2.1 Suicidal Ideation Attributes Scale

The SIDAS consists of five items—frequency, controllability, likelihood of attempt, distress, and involvement—with daily function scored based on a 10-point scale during the past month. The participants who give a score of 0 (never) to the first item should skip the rest and are given a score of 10 for controllability and 0 for the remaining items. The total scores vary from 0 to 50. The higher the scores, the greater the intensity of suicidal thoughts. The calculated cut-off point was 21, which shows the high severity of suicidal ideation. The original English version of the SIDAS showed high internal consistency and good convergent validity with the Columbia-Suicide Severity Rating Scale (22).

#### 2.2.2 Connor–Davidson Resilience Scale

The short form of the Connor–Davidson Resilience Scale (CD-RISC), which was designed based on the CD-RISC-25, includes 10 self-report items, each of them rated on a 5-point Likert scale (23). The total score of this questionnaire, which is used widely to examine resiliency and specifically the capacity to cope with difficult situations (24), ranges from 0 to 40. The higher scores show greater resiliency of the participant.

#### 2.2.3 Beck Scale for Suicide Ideation

The Beck Scale for Suicide Ideation (BSSI) includes 19 items that assess the severity of suicidal wishes and plans. Scores vary from 0 to 38; a higher score is an indicator of a higher level of suicidal ideation (25). If the individuals give a score of 1 or 2 to the fifth item, they will answer the rest of the items; otherwise, the questionnaire will not be answered. There is no cut-off point to classify the grades (26). The validity and reliability of the BSSI in English have been investigated frequently, and Cronbach's alpha coefficient was almost always above 0.85 (27–29). The reported Cronbach's alpha for both parts of the Iranian version was satisfactory (>0.8) (30).

#### 2.2.4 Beck Hopelessness Scale

It is a self-reported questionnaire consisting of 20 items, which is developed to evaluate negative expectations about the future (31). The total hopelessness score is obtained from the sum of items (range 0–20) (32). Total scores of 0 to 3 are considered normal, 4 to 8 mild depression, 9 to 14 moderate depression, and more than 14 severe depression. The reported Cronbach's alpha coefficient for the Iranian version was 0.87 (33).

### 2.3 Analysis

SPSS-29 was used for the analysis of data. In order to assess the face and content validities of the Persian version of the SIDAS (SIDAS-P), the CVI index was calculated. Correlations between the SIDAS and Beck Hopelessness Scale (BHS) and BSSI total scores were used to assess the convergent validity of the SIDAS. Similarly, in order to examine the convergent validity correlations, the SIDAS total score and the CD-RISC were calculated. Factor analysis was also calculated to assess whether the SIDAS items measured a single construct. The internal consistency of the SIDAS scale was evaluated using Cronbach’s alpha. For the purpose of confirming the factors of the SIDAS, confirmatory factor analysis was performed using the AMOS software. Eventually, receiver operating characteristic (ROC) curves were used to evaluate whether this scale could recognize individuals at high risk from those at a lower risk for suicidality.

## 3 Results

Out of 398 participants in this study, 286 (71.9%) were women. The average age of the participants was 23.9 years. The educational level of most of the participants was a bachelor's degree (72.9%), and the associate degree had the lowest number of participants (1%) (**Table 1**).

**Table 1:** The sample demographic characteristics and SIDAS total scores for subgroups of the full sample.

	N (%)	Mean (SIDAS score)	SD (SIDAS score)
age groups			
18–25	311 (78.1)	5.73	9.3
26–35	65 (16.3)	3.44	7.76
36–50	22 (3.8)	1.18	3.82
Gender			
Female	286 (71.6)	5.41	8.76
Male	112 (28.4)	4.34	9.32
Education			
Associate degree	4 (1)	0	0
Bachelor	290 (72.9)	5.84	9.28
Master	51 (12.8)	4.11	8.48
PhD	38 (9.5)	2.97	7.80
MD	10 (2.5)	1.60	4.06
Residency	5 (1.3)	0	0

SIDAS, Suicidal Ideation Attributes Scale.

The calculated score of the CVI index is a number between 0 and 1. Acceptance of the item is based on the fact that a score higher than 0.79 is suitable, a score between 0.70 and 0.79 needs to be revised, and a score lower than 0.70 is unacceptable. Considering that the lowest score was 0.92 (**Table 2**), the face validity of all questions of the SIDAS scale is suitable in terms of clarity and fluency.

**Table 2:** Face validity of the Persian version of the Suicidal Ideation Attributes Scale (SIDAS).

No.		CVI (Clarity)	CVI (Fluency)
1	In the past month, how often have you had thoughts about suicide?	1	1
2	In the past month, how much control have you had over these thoughts?	1	0.92
3	In the past month, how close have you come to making a suicide attempt?	1	0.92
4	In the past month, to what extent have you felt tormented by thoughts about suicide?	1	0.92
5	In the past month, how much have thoughts about suicide interfered with your ability to carry out daily activities, such as work, household tasks, or social activities?	0.92	0.92

The content validity of the SIDAS-P was also examined in the two dimensions of relevance and coverage. The results of the CVI index indicate that the content validity of all questions of the SIDAS was appropriate, and the lowest score was 0.83 (**Table 3**).

**Table 3:** Content validity of the Persian version of the Suicidal Ideation Attributes Scale (SIDAS).

No		CVI (Relevancy)	CVI (Coverage)
1	In the past month, how often have you had thoughts about suicide?	0.83	1
2	In the past month, how much control have you had over these thoughts?	1	1
3	In the past month, how close have you come to making a suicide attempt?	1	1
4	In the past month, to what extent have you felt tormented by thoughts about suicide?	1	1
5	In the past month, how much have thoughts about suicide interfered with your ability to carry out daily activities, such as work, household tasks, or social activities?	1	1

### 3.1 Convergent validity

Pearson's correlation test showed that there was a statistically significant relationship between the SIDAS-P and the BSSI (p < 0.01, n = 398, and r = 0.533), and the intensity of the obtained correlation was moderate. Also, the correlation between the SIDAS-P and the Beck Hopelessness Scale showed that there was a weak correlation between these two tests.

### 3.2 Divergent validity

The results of Pearson's correlation test showed that there was a negative correlation between the SIDAS and the Connor–Davidson Resilience Scale (p < 0.01, n = 398, and r = −0.416), and the severity of the obtained correlation was moderate.

The test–retest reliability was also investigated. Pearson's correlation coefficient test showed that there was a significant correlation between the test and retest results (p < 0.01, n = 50, and r = 0.575), and the intensity of this correlation was moderate.

Furthermore, Cronbach's alpha was used to check the internal consistency of the SIDAS-P. The obtained alpha coefficient was 0.799, which is at an acceptable level.

### 3.3 Confirmatory factor analysis

**Figure 1:**
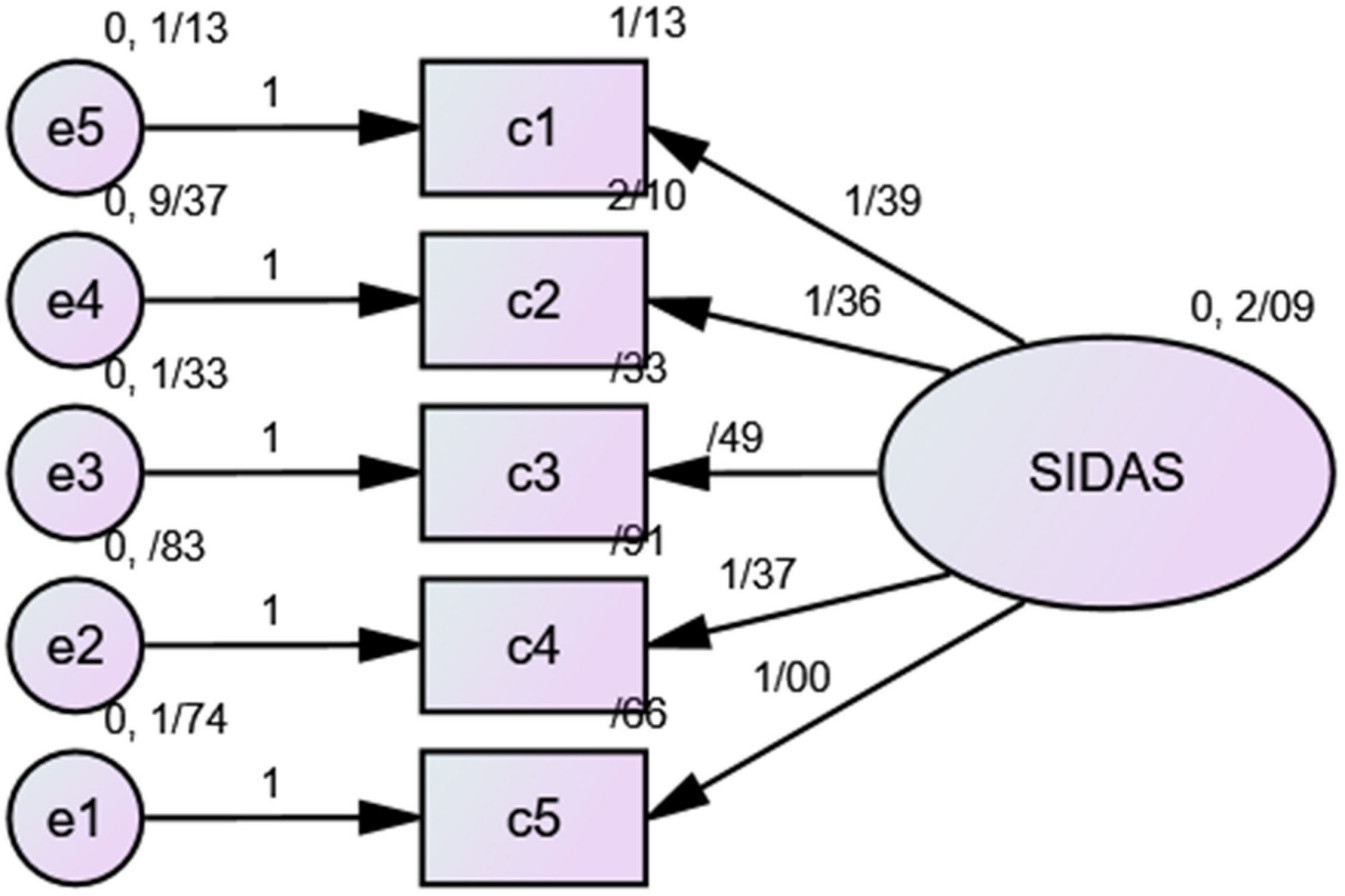
Confirmatory factor analysis of SIDAS. SIDAS, Suicidal Ideation Attributes Scale.

Confirmatory factor analysis (CFA) was conducted to investigate the unifactorial structure of the SIDAS (**Figure 1**).

As shown in **Table 4**, all factor loadings were higher than 0.4 and were significant. The t-statistic or critical ratio (CR) was also greater than 1.96.

**Table 4:** Regression of SIDAS.

			Estimate	SE	CR	p
c5	<---	SIDAS	1/000			
c4	<---	SIDAS	1/374	0/077	17/752	0/000
c3	<---	SIDAS	0/491	0/048	10/146	0/000
c2	<---	SIDAS	1/356	0/130	10/464	0/000
c1	<---	SIDAS	1/387	0/079	17/449	0/000

SIDAS, Suicidal Ideation Attributes Scale; CR, critical ratio.

According to the results of the goodness-of-fit index table, the values of comparative fit index (CFI), Incremental Fit Index (IFI), and normed fit index (NFI) were all higher than 0.9, which indicates a very good fit. The chi-squared test was 891/59, which is suitable, but minimum discrepancy of confirmatory factor analysis/degrees of freedom (CMIN/DF) is higher than the conventional value of 3 (**Table 5**).

**Table 5:** Fit indices of the single-factor model of the SIDAS.

CFI	IFI Delta2	NFI Delta1	RMSEA	CMIN/DF	Df	CMIN	p
0/943	0.943	0.938	0.166	11.978	5	59.891	0.000

GFI, the Incremental Fit Index; NFI, normed fit index; minimum discrepancy of confirmatory factor analysis/degrees of freedom, CFI, comparative fit index; RMSEA, root mean square error of approximation.

### 3.4 ROC curve analysis

**Figure 2:**
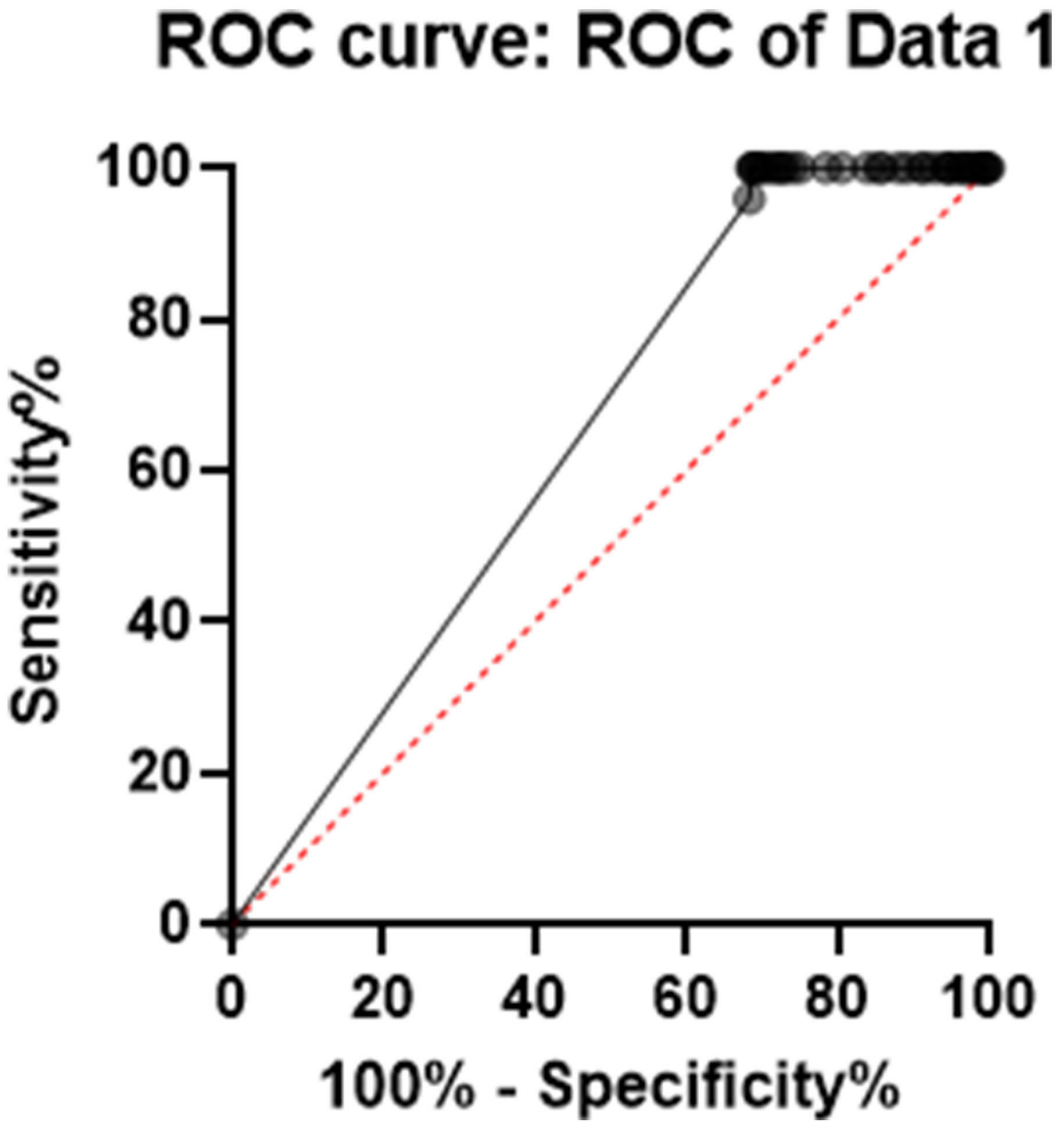
Predictive power of the SIDAS. SIDAS, Suicidal Ideation Attributes Scale.

This analysis was used to ascertain whether the SIDAS-P was good enough in recognizing participants classified above the low risk. For this purpose, question 6 of the BSSI was used. Therefore, those who gave a rate of 2 or 3 to this item were selected as the people with above the low risk of suicide. The optimal cut-off point was the highest sensitivity and the lowest specificity, and according to the results, scores higher than 6.5 on the SIDAS are above the low risk for suicide. The accuracy index of the evaluation scale is the area under the curve, which was obtained in this test [area under the curve (AUC) = 0.909] (**Table 6**), and therefore, the test's detection power is at an excellent level (**Figure 2**).

**Table 6:** Predictive power of the SIDAS for moderate suicide risk.

The area under the curve				
Confidence interval		p	SE	Area under the curve
Upper limit	Lower limit			
0.873	0.560	0.003	0.080	0.717

SIDAS, Suicidal Ideation Attributes Scale; CR, critical ratio.

## 4 Discussion

The current study examined the psychometric properties of the Persian version of the SIDAS with a sample of Iranian college students. In the present study, as the first step, in order to check the convergent validity based on previous studies, the Beck Scale for Suicide Ideation and Beck Hopelessness Scale were used, and Pearson's correlation indicated a moderate degree of correlation with the Beck Scale for Suicide Ideation and a weak degree of correlation with the Beck Hopelessness Scale. Moreover, the divergent validity was assessed using the CD-RISC. As noted previously, the results of Pearson's correlation test indicated a negatively moderate correlation, suggesting that the SIDAS does not measure any other construct except suicide. In terms of reliability, the SIDAS-P demonstrates high internal consistency, which is consistent with other studies conducted in different countries and populations (18, 19, 34).

Furthermore, ROC curves were used in order to evaluate whether the SIDAS could recognize the participants classified above the low risk for suicide by assessing scale sensitivity and specificity.

To explain the low correlation between the SIDAS scores and BHS scores, its founders, Beck and colleagues, reported that the BHS can predict suicide in a sample of outpatients and inpatients (35). However, there are some doubts about the utility of the BHS. Some studies have not shown the effectiveness of the scale in successfully predicting suicide or self-harm (36). Furthermore, evidence indicated that the BHS is usually less reliable in non-psychiatric populations because they generally obtain lower levels of hopelessness, while this scale was originally developed for clinical aims and based on a clinical sample consisting of suicide attempters (37, 38). A meta-analysis showed that the BHS has low predictive values for suicide, with a sensitivity of 29% to 54% and a specificity of 60% to 84% (37). Furthermore, a study on depressed individuals indicated that participants’ scores on beck depression inventory (BDI) predicted their future suicide attempts, but not hopelessness severity (BHS) (38). It is also noteworthy to acknowledge that the SIDAS just assesses the active suicidal ideation and not the passive one like the BHS (e.g., feeling that life is not worth living), which can be an important indication for the low calculated correlation. Considering all the above, it seems that the BHS is not a suitable choice as a convergent validity scale, and future researchers are suggested to use tools specifically designed for screening and assessing suicide.

Although the SIDAS is a brief, web-based scale and economical in terms of time, it is possible that other characteristics that are more closely related to the severity of thoughts and a higher likelihood of suicide were not included in the current scale (unlike the BSSI). In other words, items such as preparations, having a plan, previous attempts, and duration of thoughts have been shown to be related to suicide but were not included in the SIDAS for brevity (22). Additionally, the view that passive suicidal ideation is clinically less serious than active one leads to giving less importance to such thoughts in assessing the risk of suicide. The National Action Alliance for Suicide Prevention has raised the view that passive ideation may be comparable to active ideation in predicting negative mental health outcomes, including suicidal behavior (39).

Although screening tools are very helpful in identifying at-risk individuals, considering that suicide is a complicated problem that is affected by multiple factors, it is essential to provide a comprehensive patient evaluation that gathers information on medical history, biographical factors, psychiatric history, family history, and social support resources in order to make final decisions regarding the patient's risk for suicide and treatment plans.

### 4.1 Limitation

Although this study is the first to examine the validity and reliability of the SIDAS questionnaire among Iranian college students, it should be considered in light of the following limitations. One of the limitations was the disproportionate representation of young individuals and women in the sample, which led to a heterogeneous sample and made the generalization of the findings difficult. Another limitation is related to the sampling method. It is suggested that future studies use random sampling. Additionally, individuals with suicidal thoughts may have chosen not to complete the questionnaires, leading to a significant portion of the sample consisting of individuals without such thoughts. Therefore, future studies could focus specifically on those experiencing suicidal ideation. Another limitation to note was the low number of participants for evaluation of reliability, which may have contributed to the low reliability of the questionnaire. The other issue that should be addressed in future studies is the low number of students in the current study's test–retest reliability, which was due to the unwillingness or unavailability of participants after a 4-week interval. Finally, it is suggested that more studies be conducted to evaluate the clinical applicability of this scale in different clinical populations in the future.

## Data Availability

The raw data supporting the conclusions of this article will be made available by the authors, without undue reservation.
